# Matrix Metalloproteinases in Health and Disease in the Times of COVID-19

**DOI:** 10.3390/biom12050692

**Published:** 2022-05-11

**Authors:** Carlos Fernandez-Patron, Eugenio Hardy

**Affiliations:** 1 Department of Biochemistry, Faculty of Medicine and Dentistry, University of Alberta, Edmonton, AB T6G 2H7, Canada; 2 Center of Molecular Immunology, P.O. Box 16040, Havana 11600, Cuba

Much has been written about matrix metalloproteinases (MMPs) in health and disease conditions, but their roles in the setting of COVID-19 and associated illnesses remain understudied. This gap in knowledge was the main motivation for the present Special Issue in Biomolecules. MMPs are a large family of enzymes that share a basic domain architecture consisting of an N-terminal propeptide that keeps the enzyme in a latent form until activated by proteolytic cleavage; a C-terminal catalytic domain that contains zinc and calcium ions and is crucial for the proteolytic activity of MMPs; and a hemopexin-like C-terminal domain that mediates substrate binding and/or interactions with a family of tissue inhibitors of metalloproteinases (TIMPs) [1,2]. Several other moieties modify this basic structure, such as a signal peptide for secretion out of the cell, the fibronectin type-II-like domain which is unique to MMP-2 and MMP-9, a hinge region that connects the catalytic domain to the hemopexin-like domain or a recognition motif for furin-like convertases located at the end of the propeptide domain and C-terminal putative transmembrane domain in most of the membrane-type MMPs [1,2]. The activity of MMPs is regulated at multiple levels including inhibition by many endogenous molecules (e.g., TIMPs, α2-macroglobulin and fibrinogen), transcription in response to a variety of factors (e.g., growth factors, physical stress, cytokines), post-transcription (e.g., glycosylation), activation by elimination of the propeptide, and compartmentalization/localization [2]. The fine regulation of MMPs activity enables many biological processes that occur under physiological conditions (embryogenesis, wound healing, or angiogenesis) and if perturbed can exacerbate the development of pathological processes (cancer, obesity, diabetes, cardiovascular diseases, arthritis, and pulmonary fibrosis [2]). Interestingly, individuals with these pathological conditions (i.e., comorbidities) have been found to be more susceptible to developing severe COVID-19 illnesses [3].

Therefore, we designed this Special Issue to advance what is currently known as well as new emerging ideas on how MMPs activity impacts diseases (in particular, COVID-19) with attention to five relevant topics.

**1.**  **MMPs in Hypertension**

The picture about the way imbalanced vascular MMP activity results in vascular remodeling in hypertensive individuals is far from being complete. Based on a mixture of established works, recent reports and their own research, a review article by Prado et al. [4] discusses about mechanisms (such as increased oxidative stress which impairs nitric oxide bioavailability and increases vascular MMP activity) that contribute to endothelial dysfunction and vascular remodeling in hypertension. They also debate how vascular remodeling could be prevented by MMP inhibition using not only MMP inhibitors but also repurposing a variety of commonly used drugs (e.g., nebivolol which is an antagonist of β1-adrenergic receptors) that may attenuate MMP activation.

**2.**  **MMP-9 in Heart Infarct**

To devise new therapies that accelerate resolution or stimulate cardiac repair, new knowledge that unravels the complex role of MMP-9 in cardiac wound healing is required. Becirovic-Agic et al. [5], in a review article, discusses in great detail the involvement of MMP-9 in all phases (such as inflammatory, proliferation and maturation) of cardiac wound healing, and provide reasonable explanations as to why MMP-9 inhibition has been unsuccessful. Given the detrimental or favorable effect of MMP-9 on cardiac wound repair, this review article emphasizes the necessity of targeting MMP-9 only for its destructive activities.

**3.**  **Aberrant MMP Activity Caused by Epigenetic Mechanisms**

How a variety of seemingly unrelated diseases (such as atherosclerosis, arthritis, tuberculosis, viral infections (including COVID-19), and cancer) and their prescribed medicinal drugs exert epigenetic pressure on the expression of MMP genes? Sarker et al. [6], in another review article, discuss how human pathologies associated with deregulated MMP activity and their commonly prescribed drugs (such as doxycycline and statins) may exert epigenetic pressure on MMP genes. Research remains to be carried out in order to understand how epigenetic mechanisms involving chemical modifications such as methylation, acetylation or phosphorylation on DNA, histones, and expression of miRNAs that affect the gene expression and resulting activity of MMPs could be exploited for prognosis, diagnosis, or drug development.

**4.**  **MMPs in Lung Disorders and COVID-19**

The latest models [7,8,9] suggest that the entry of SARS-CoV-2 into target cells (such as type II alveolar epithelial cells) involves a specific binding of spike (S) glycoprotein of coronavirus to the angiotensin-converting enzyme 2 (ACE2) receptor located on the host cell surface. The entry process is mediated by a variety of host cell enzymes including the surface serine protease 2 (TMPRSS2) which cleavages the viral S-protein, endosomal cathepsin proteases (e.g., Cat L and Cat B), trypsin and furin. The fusion of SARS-CoV-2 virus with the host cell membrane followed by virus endocytosis induces the release of endosomal cathepsin proteases (e.g., Cat L and Cat B). The release of viral RNA into the cytoplasm and subsequent rapid production of new viral particles which are secreted by exocytosis has pathological consequences. A specific immune response is concomitantly activated to eliminate the SARS-CoV-2 and start the inflammatory response. Innate immune cells (e.g., macrophages) detect the virus and secrete pro-inflammatory mediators such as chemokines, cytokines, and growth factors. Cytokines activate and attract more immune cells in the infection site (e.g., lung tissue), which then produce more cytokines (i.e., the so-called “cytokine storm”) generating an unrestrained cycle of inflammatory response. Secretion of MMPs from activated immune and non-immune cells is then increased in the inflamed sites, which consequently leads to extracellular matrix remodeling and massive tissue damages. Damages associated with the cytokine storm are the disequilibrium of the MMPs/TIMPs ratio, increase deposition of fragments generated by extracellular matrix disruption, and substantial fibrin deposition. These events may lead to pulmonary endothelial damage, several diseases (such as pulmonary edema and acute respiratory distress syndrome) and organ failure, which may result in death.

Several recent reports describe the involvement of specific MMPs in COVID-19. MMP-1 together with other factors (e.g., vascular endothelial growth factor-A) has been found elevated in hospitalized SARS-CoV-2-infected patients and also associated with the severity of COVID-19 [10]. MMP-2 has been observed to be downregulated and the plasmatic levels of MMP-2 correlate with mortality in severe COVID-19 disease, being therefore proposed as a prognostic predictor for risk of in-hospital death caused by coronavirus disease-2019 [11]. Serum MMP-3 level has been proposed as a valuable marker of COVID-19 in SARS-CoV-2 infected subjects, and may also be associated with COVID-19 severity [12,13]. Circulating levels of MMP-7 has been reported as a potential biomarker of COVID-19 severity to discriminate patients requiring mechanical ventilation. Together with other circulating biomarkers (such as the programmed death-ligand 1 and T cell immunoglobulin and mucin domain-3) and in combination with other set of data (e.g., clinical and hemodynamic), circulating MMP-7 could help physicians decide when to proceed with intubation. MMP-7 could also serve as an indicator of lung lesions post-COVID-19 [14]. MMP-9 has been observed upregulated and suggested as an early marker of respiratory failure in severe COVID-19 patients [11,15]. Also, in conjunction with other parameters (high viral load and weak antibody response), MMP-9 has been associated with persistent pulmonary pathology after COVID-19 [16]. In the absence of membrane-bound serine proteases, MMP-2 and MMP-9 may act by facilitating SARS-CoV-2 entry in cells expressing high MMP levels [17]. MMP-2, MMP-3, MMP-9, and MMP-12 in conjunction with other molecules (cytokines, chemokines and adhesion molecules) are thought to be potentially involved in the development of COVID-19-induced neurological symptoms [18]. High serum levels of MMP-7 and MMP-9 jointly with other two pro-fibrotic markers (platelet-derived growth factor and transforming growth factor-β) have been found to be associated with the severity of COVID-19 in obese-diabetic patients [19]. Last but not least, an original research article by da Silva-Neto et al. [20] is aimed at advancing our understanding of how MMPs function in severe COVID-19 lungs, by measuring and analyzing the expression and activity of MMPs in the lung tissue microenvironment including samples from intubated COVID-19 and non-COVID-19 patients. They found a positive correlation between the activity of upregulated MMP-8 and MMP-2 with the immune response associated to the shedding of immunosuppression mediators (soluble human leukocyte antigen-G and triggering receptor expressed on myeloid cells 1), and suggest that COVID-19-caused severe lung damage could be promoted by the concerted action of inflammatory response and oxidative stress together with overexpression of the MMP-2/MMP-8 axis.

**5.**  **MMPs in the Development of Disease Tolerance**

An interesting question concerns whether the target of therapies to fight SARS-CoV-2 infection (and other pathogens) should be (a) the pathogen itself, (b) the pathological effects caused by the pathogen-host interaction, or (c) a combination of both? After recapitulating the roles played by MMPs in pulmonary pathologies (e.g., acute lung injury and acute respiratory distress syndrome) and the development of COVID-19, Hardy and Fernandez-Patron [21] bring attention to the idea MMPs may play roles in the modulation of host tolerance to disease (including COVID-19) and their activity is thus likely to impact the effectiveness of traditional pathogen-specific therapeutic approaches such as vaccines, antivirals or antimicrobials.

In short, this Special Issue combines established knowledge and new emerging insights on how MMPs activity affects the transition from health to disease, with a focus on the development of COVID-19 illnesses and associated comorbidities.

## Data Availability

Not applicable.
